# Vasculitis and Systemic Lupus Erythematous (SLE)

**DOI:** 10.1186/1824-7288-41-S2-A20

**Published:** 2015-09-30

**Authors:** Elisabetta Cortis, Maria Greca Magnolia

**Affiliations:** 1 Referrall Centre for Paediatrics Rheumatology, Orvieto, Italy

## 

Vasculitis are a heterogeneous group of disorders characterized by inflammation of the blood vessels of different caliber and sometimes fibrinoid necrosis with vessel wall destruction [1].

Vasculitis are divided into cutaneous and systemic forms, primary and secondary to hypertension, immunosuppression therapy, metabolic complications. The classification is based on the affected vessel size (Table 1). They may have neurological manifestations at the onset and during the desease development. These are more common in systemic forms such as SLE and Nodose Polyarteritis (PAN).

**Table 1:** **Vasculitis: classification** (Adapted from EULAR/PReS endorsed criteria for the classification of childhood vasculitis) 2006; 65:936-41

**Vasculitis in large calibre vessels prevalence** • Takayasu arteritis **Vasculitis in medium caliber vessels prevalence** • Nodose Polyarteritis • Cutaneous polyarteritis • Kawasaki desease **Vasculitis in small calibre vessels** A.Granulomatous • Wegener Granulomatosis • Churg-Strauss syndrome B. Non granulomatous • Microscopic polyangiitis • Schönlein-Henoch syndrome • LVC isolated • Urticarial vasculitis ipocomplementemica **Other forms** • Behcet desease • Vasculitis secondary to infection (including nodose polyarteritis associated with hepatitis B), in tumor sand infections, including hypersensitivity vasculitis) • Connective tissue diseases associated vasculitis • Isolated SNC vasculitis • Cogan syndrome • Not classified vasculitis

The Schonlein-Henoch purpura and Kawasaki disease, the most frequent vasculitisin childhood, rarely can have neurological disorders. There are forms of mild to moderate intensity like headache, irritability, mood disorders and behavioral and forms of severe as seizures and sensory disturbance up to coma.

In the course of SLE, neuropsychiatric manifestations, headache and chorea are common, with an incidence of 20-40% (also 80% with cognitive disorders and asymptomatic alterations RMN). The neuropsychiatric manifestations involve 40-56% of children; headache the 22-64%; convulsions the 20-31%; chorea 4-10%; peripheral neuropathy 5-6%; myelopathy 1%. Heterogeneity in their neurological symptoms are important for prognostic purposes.

In antiphospholipid syndrome, primary or secondary, the following are common: transient cerebral ischemia and ischemic stroke, memory loss, chorea, seizures, vision problems [2].

The PAN is a necrotizing vasculitis histo-pathological examination, rarely aneurysm, stenosis or occlusion (not caused by fibro-muscular dysplasia, or by other causes not inflammatory) artery of small and medium caliber. In addition, at least one of the following signs/symptoms: skin involvement (livedo, nodules or heart attacks); myalgia; hypertension; peripheral neuropathy (sensory or motor); renal involvement (proteinuria, haematuria, renal impairment).

The primary central nervous system vasculitis is a brain vessels inflammation not associated with vasculitis of other organs. The classification is based on the vessel size: small (with normal angiography) and medium-large (progressive and non-progressive). This form, responsible for 40-60% of arterial ischemic stroke, affects 3-8/100.000 children/year. Symptoms are characterized by acute severe headache (80%), focal neurological deficit (78%), motor deficit (62%), cognitive disorders (54%), cranial nerve involvement (59%), seizures (small vessel vasculitis).

Peripheral neuropathies are characteristic of the Churg-Strauss disease.

The clinical diagnosis is often difficult (Table 2).

**Table 2:** Vasculitis: diagnosis

• **clinical sympthoms:** are not specific (fever, myalgia, fatigue, asthenia, bone and joint pain, weight loss) at the onset • **laboratory tests**: are not specific tests expept cANCA in Wegner disease and pANCA in microscopic polyangioitis; Ag factor VIII Won Willebrand, soluble selectins, show endothelial damage of the blood vessels • **instrumental tests**: echocardiogram “coronary dilatation typical of KD” also foundins JIA; MRI angiography alternative “safe” for TA diagnosis but not for the studyof small caliber vasculitis • **histo-pathological examination**: it may help in the diagnostic confirmation

Neurological complications are diagnosed early because the treatment must be immediate and aggressive.
